# Effects of protein content and the inclusion of protein sources with different amino acid release dynamics on the nitrogen utilization of weaned piglets

**DOI:** 10.5713/ab.21.0142

**Published:** 2021-08-21

**Authors:** Nianzhi Hu, Zhiwen Shen, Li Pan, Guixin Qin, Yuan Zhao, Nan Bao

**Affiliations:** 1 Key Laboratory of Animal Production, Product Quality and Security, Ministry of Education, Jilin Provincial Key Laboratory of Animal Nutrition and Feed Science, College of Animal Science and Technology, Jilin Agricultural University, Changchun 130118, China

**Keywords:** Amino Acid Release Dynamics, Dietary Protein Content, Dietary Protein Source, Piglet

## Abstract

**Objective:**

We aimed to investigate the effect of the differing amino acid (AA) release dynamics of two protein sources on the growth performance, nitrogen deposition, plasma biochemical parameters, and muscle synthesis and degradation of piglets when included in their diets at normal and low concentrations.

**Methods:**

Forty-eight piglets (Duroc×Landrace×Large White) with initial body weight of 7.45±0.58 kg were assigned to six groups and fed one of 6 diets. The 6 dietary treatments were arranged by 3×2 factorial with 3 protein sources and 2 dietary protein levels. They are NCAS (a normal protein content with casein), NBlend (a normal protein content with blend of casein and corn gluten meal), NCGM (a normal protein content with corn gluten meal), LCAS (a low protein content with casein), LBlend (a low protein content with blend of casein and corn gluten meal), LCGM (a low protein content with corn gluten meal). The release dynamics of AA in these diets were determined by *in vitro* digestion. The digestibility, utilization and biological value of nitrogen in piglets were determined by micro Kjeldahl method. Plasma insulin was measured by enzyme-linked immunosorbent assay kits. The protein expression of mediators of muscle synthesis and degradation was determined by western blotting.

**Results:**

Although the consumption of a low-protein diet supplemented with crystalline AA was associated with greater nitrogen digestion and utilization (p<0.05), the final body weight, growth performance, nitrogen deposition, and phosphorylation of ribosomal protein S6 kinase 1 and eIF4E binding protein 1 in the muscle of pigs in the low-protein diet-fed groups were lower than those of the normal-protein diet-fed groups (p<0.05) because of the absence of non-essential AA. Because of the more balanced release of AA, the casein (CAS) and Blend-fed groups showed superior growth performance, final body weight and nitrogen deposition, and lower expression of muscle ring finger 1 and muscle atrophy F-box than the CGM-fed groups (p<0.05).

**Conclusion:**

We conclude that the balanced release of AA from CAS containing diets and mixed diets could reduce muscle degradation, favor nitrogen retention, % intake and improve growth performance in pigs consuming either a normal- or low-protein diet.

## INTRODUCTION

The shortage of high-quality nitrogen resources and the environmental pollution caused by fecal and urinary nitrogen loss have always been challenges in pig production [1]. In particular, although diets containing high levels of crude protein (CP) promote the normal development of weaned piglets, they are associated with the above problems and with the risk of intestinal diseases in piglets [2]. A series of previous studies have shown that piglets fed a diet with a low CP content (no more than 3% unit less than standard) that is supplemented with free amino acids (FAA) lose less nitrogen and are cheaper to feed, and they demonstrate appropriate growth performance and have a lower risk of developing gastrointestinal tract (GIT) diseases [3–5].

The final degradation products of intact protein in the GIT are FAA and peptides [6], which enter intestinal capillaries and are transported to the liver for protein synthesis. Thus, the utilization of protein by animals is related with utilization of amino acids (AA). In the current feed evaluation system [7], the nutritional needs of pigs are estimated according to the composition and ileal terminal digestibility of AA. However, the digestion and absorption of feed materials in the GIT of animals is a dynamic process that is not reflected in these parameters [7].

Previous study has shown that the consumption of whey protein, which is rapidly digested, leads to earlier postprandial hyperaminoacidemia and peak protein synthesis because most of the AA are released quite proximally in the GIT, which means that FAA enter the portal circulation rapidly [8]. However, this will also result in excessive degradation of high-quality AA (i.e., essential amino acids, EAA) in the GIT, and it comes to be a waste of nitrogen. Conversely, the consumption of casein (CAS), which is digested more slowly, is associated with a shallower protein synthesis peak and less protein degradation in the GIT, such that the total protein deposition is higher than that achieved following the consumption of whey protein [9]. Study suggested that a mixture of proteins (fast and slow AA-releasing) provides the advantages of both types because the rapid appearance and maintenance of a high concentration of AA prolong hyperaminoacidemia and increase protein retention [10].

The incubation of diets with pepsin and trypsin has been shown to be an effective means of simulating *in vivo* digestion in the porcine GIT [11]. In addition, the dynamics of the digestion of diets can be reflected to some extent by measuring the FAA concentrations in the *in vitro* residue at various time points [12]. On the basis of such studies, we have shown that CAS releases its AA faster than soybean meal (SBM) [6], and SBM releases its AA faster than corn gluten meal (CGM) [13]. In the present study, CAS or CGM were included in six diets at two kinds of CP levels (normal or low), and we aimed to i) determine their AA release dynamics *in vitro*, and ii) identify the relationship between the dynamics of AA release and nitrogen deposition in piglets consuming the diets by measuring their growth performance, blood biochemical indices, nitrogen balance, and protein expression of mediators of muscle synthesis and degradation. The overall objective of the study was to identify the optimal ways of using nitrogen resources, reducing production costs and protecting the environment in pig production.

## MATERIALS AND METHODS

### Animal care

This study was approved by the Animal Care and Use Committee of Jilin Agricultural University (process number: KT2019012). The experimental procedures, including animal care, were managed in strict accordance with the “Regulations on the Management of Experimental Animals (November 17, 2016)” issued by Jilin Provincial People’s Government.

### Animals, housing, experimental design and diet

Forty-eight hybrid piglets (Duroc×Landrace×Large White) with an initial body weight of 7.45±0.58 kg were allocated to 6 treatments of 4 replicates each with 2 pigs per replicate, according to their body mass and sex in a completely randomized design. Treatments were designed by a 2×3 factorial arrangement of treatments, and the main factors were 2 different CP levels and 3 different protein sources of the diet.

The experimental diets contained either a normal protein concentration (CP 17.5% standardized ileal digestible, SID) or a low protein concentration (CP 14.8%, SID). Three diets were prepared in each category according to the release rate of AA: a CAS-based diet, a CGM-based diet, and a diet containing a mixture of the two (CAS+CGM; Blend). The diets were formulated (Table 1) to meet the estimated nutrient requirements for piglets [7]. The overall concentrations of various AA in each diet were balanced by adding crystalline amino acids (CAA) and the other components of the low-protein diets were present at similar concentrations to those in the normal-protein diets.

The study was performed in the livestock housing of Jilin Agricultural University, which is maintained at a temperature of 25°C. Piglets were fed three times a day, at 0700, 1200, and 1800, such that a small amount of feed remained each time. They had free access to water from teat dispensers. The piglets were permitted to adapt to their environment for 5 days, then they were fed the diets for 28 days, and their daily feed intake and body weight were measured at the start and end of the trial, on days 6 and 33. These values were used to calculate the average daily feed intake (ADFI), average daily gain (ADG), and gain-to-feed ratio (G:F).

### Determination of the amino acid release rates for each diet

#### In vitro digestion experiment

Following the methods of Bai et al [12] and Abdallah et al [13] which was modified from the method developed by Boisen and Fernández [11], 1 g of each feed sample (measured to 0.001 g) was placed in 100 mL conical flasks, with three replicates per diet. Ten milliliters of freshly prepared 1 mg/mL pepsin (pH 2) and 0.5 mL chloramphenicol solution (0.5 g chloramphenicol in 100 mL ethanol) were added to the conical flasks, which were sealed and incubated in a water bath oscillator at 39°C for 4 h. At the end of the incubation, the conical flasks were removed, 10 mL phosphate buffer (pH 6.8) was added, and the pH was adjusted to 6.8 using 1 M NaOH. One milliliter of 50 mg/mL trypsin solution was then added, the contents were mixed, and the flasks were re-covered with sealing membrane and incubated at 39°C for a further 0, 4, 8, 12, 16, 20, 24, and 28 h. The solutions were centrifuged at 2,000×g and 4°C for 15 min, 800 μL of each supernatant were passed through 0.22 μm filters, 200 μL 10% sulfosalicylic acid was added, and the mixtures were incubated at 4°C for 1 h and then centrifuged at 8,000 ×g for 10 min. The supernatants were then filtered through 0.22 μm filters and stored at −80°C until analysis.

#### Chromatographic conditions

As previously described by Abdallah et al [13], an Acquity ultra-high performance liquid chromatography (UPLC) tunable ultraviolet system (Waters, Milford, MA, USA) was used. The chromatographic column was an AccQ·Tag Ultra column (2.1×100 mm, P/N: 186003837), mobile phase A was 10% AccQ·tagUltra eluent A, mobile phase B was AccQ·tagUltra eluent B, the flow rate was 0.7 mL/min, the injection volume was 1 μL, the column temperature was 55°C, the sample temperature was 15°C, the detection wavelength was 260 nm, the collection speed was 20 points/s, the time constant was 0.1 s, and the running time was 10 min.

#### Pre-column derivation

One milliliter of AccQ fluor diluent from the 2B bottle was injected into the 2A (AccQ Fluor reagent powder) bottle, vortexed, and incubated at 55°C for 10 min. Seventy milliliters of AccQ·Fluor·Buffer 1 and 10 mL of sample were added to derivative tubes (P/NWT007571), followed by 20 mL of AccQ Fluor derivative (2A), and the tubes were mixed for 15 s and placed in a 55°C oven for 10 min after standing for 1 min at room temperature. The derivatives were then transferred to UPLC full recovery sample bottles.

#### Creation of free amino acid curves

Graphs of AA release during trypsin digestion were plotted using Graphpad Prism version 8.0 (GraphPad Software, San Diego, CA, USA). We considered that the AA would be almost completely released by 28 h. Therefore, the percentage FAA release was calculated by dividing the FAA concentrations at 0, 4, 8, 12, 16, 20, 24, and 28 h by those at 28 h. The absolute differences between the individual FAA release percentage curves and the total free amino acid (TFAA) release curves were then calculated.

### Nitrogen balance

After assessing the growth of the piglets, nitrogen balance was assessed from day 34 of the study. Four piglets with body weight closest to the mean values were selected from each diet group and placed into metabolic pens with slatted floor and urine collector, where they were fed two meals daily, at 0900 h and 1600 h. The nitrogen balance experimental diets were the same as the growth performance experimental diets. After 3 days of adaptation, the feces and urine of each pig were collected for 4 consecutive days. All feces were collected at 0900 h a day after feeding, weighed and frozen at −20°C. After the trial, the feces were thawed and homogenized, and 10% of the final weight of the sample was taken. The fecal sample was dried in an oven at 65°C and then crushed in high-speed multifunction crusher (FW100, Tester, Tianjin, China). Comminuted fecal sample was stored at −20°C for measurement. After feeding at 0900 h and 1600 h twice daily, the whole urine in the urine collector was removed and weighed, and subsequently filtered with gauze, taking 10% of the total weight into a clean container. Twenty mL of 2 M sulfuric acid was added to the urine collector for nitrogen fixation before each collection. After the trial, the urine samples were thawed and mixed and then frozen at −20°C for subsequent analysis. The nitrogen content of the samples was measured using a fully automatic Kjeldahl nitrogen analyzer (Foss, Hillerød, Denmark) according to Kjeldahl method [14] and the nitrogen digestibility, nitrogen retention, % intake and nitrogen biological value were calculated.


Nitrogen digestibility (%)=(nitrogen intake-fecal nitrogen)/nitrogen intake×100%

Nitrogen retention, % intake (%)=(nitrogen intake-fecal nitrogen-urine nitrogen)/nitrogen intake×100%

Nitrogen biological value (%)=(nitrogen intake-fecal nitrogen-urine nitrogen)/(nitrogen intake-fecal nitrogen)×100%

### Serum and tissue sample collection

On day 33 of the study, 10 mL blood was collected from a jugular vein of each piglet into heparinized tubes, and the separated plasma was stored at −80°C for subsequent measurements. On day 34, 2 hours after feeding at 0700 h, the piglets were anesthetized, blood samples were obtained, and samples of the *longissimus dorsi* muscle were collected and immediately transferred to −80°C for subsequent analysis.

### Determination of plasma biochemical indices

Plasma urea nitrogen (PUN) was measured using an automatic biochemical analyzer (Biobase, Shandong, China). Plasma insulin concentration was determined using enzyme-linked immunosorbent assay kits (Mbbiology, Jiangsu, China), according to the manufacturer’s instructions.

### Measurement of the expression of mediators of muscle synthesis and degradation

The protein expression of mammalian target of rapamycin (mTOR) pathway and the ubiquitin-proteasome pathway (UPP) intermediates in *longissimus dorsi* muscles was determined by western blotting. Briefly, the muscle samples were ground in mortars under liquid nitrogen and lysates were prepared in radio immunoprecipitation assay lysis buffer (strong) (Cwbio, Beijing, China) supplemented with phosphatase and protease inhibitors (Cwbio, China). The protein concentrations of the lysates were quantified using bicinchoninic acid assay protein kits (Cwbio, China). The protein concentrations of the lysates were equalized, and the lysates were mixed with loading buffer and heated in boiling water for 10 min. Polyacrylamide gel electrophoresis was performed, and then the proteins were transferred to polyvinylidene fluoride membranes (Millipore, Burlington, MA, USA). The membranes were then blocked using 5% fetal bovine serum at room temperature for 1 h and incubated overnight at 4°C with antibodies targeting phosphorylated ribosomal protein S6 kinase (p-S6K1), muscle atrophy F-box (MAFbx), muscle ring finger 1 (MuRF1) (Abclonal, Wuhan, China), or phosphorylated elF 4E binding protein-1 (p-4E-BP1) (Bios, Beijing, China). The next day, the membranes were incubated with secondary antibody at room temperature for 1 h. Polyvinylidene fluoride membranes were imaged using the Kodak gel imaging system (Kodak, Rochester, New York, USA) after reacting the membranes for approximately 1 min in the dark with electrochemiluminescence developing solution (Cwbio, China). The intensities of the specific bands were analyzed using Image J (NIH, Bethesda, MD, USA) and the relative expression of each protein was calculated.

### Statistical analysis

The data were analyzed using SPSS version 20.0 (IBM, Inc., Armonk, NY, USA). Protein content, protein source and their interaction effects were analyzed with general linear model univariate for two-way analysis of variance (ANOVA). When there was interaction between protein content and protein source, means of the six treatments were analyzed by multiple comparisons using the LSD method of one-way ANOVA. The data are expressed as means and standard errors of the mean (SEM). Each pen was the experimental unit. Statistical significance was accepted at p<0.05.

## RESULTS

### Release dynamics of amino acids

**Figure 1:**
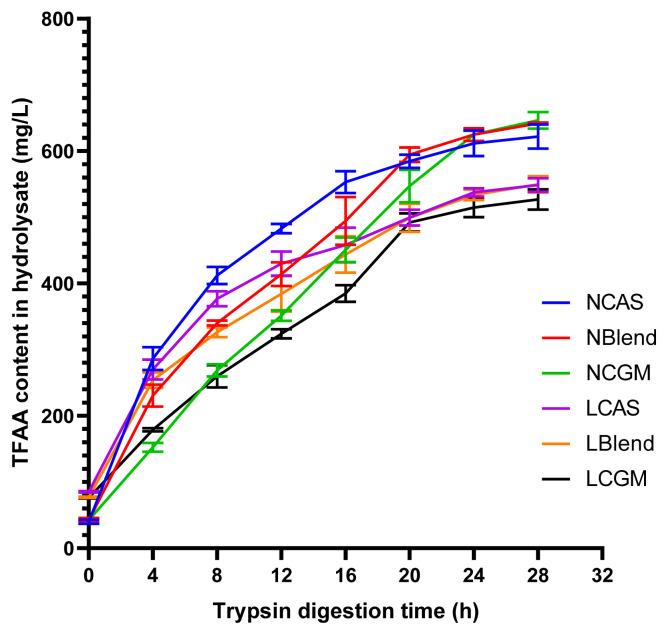
Total free amino acid release from each diet over time. NCAS, diet with normal protein content, based on casein; NBlend, diet with normal protein content, based on casein and corn gluten meal; NCGM, diet with normal protein content, based on corn gluten meal; LCAS, diet with low protein content, based on casein; LBlend, diet with a low protein content, based on casein and corn gluten meal; LCGM, diet with low protein content, based on corn gluten meal; TFAA, total free amino acid concentration.

**Figure 2:**
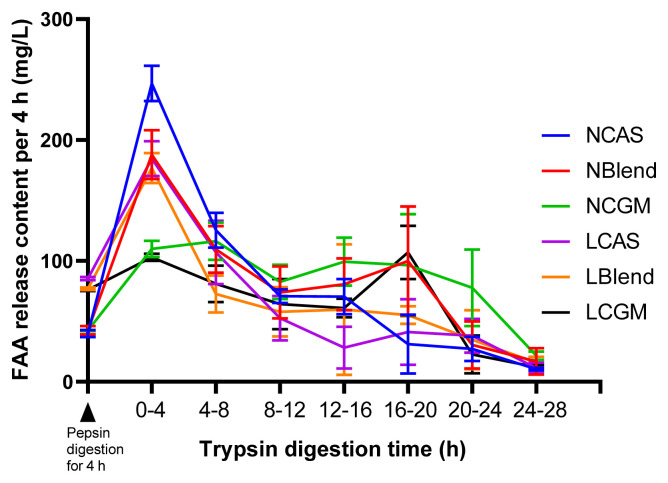
Free amino acid release from each diet during 4 h time intervals. At the zero time point, the diet had been digested using pepsin for 4 h. NCAS, diet with normal protein content, based on casein; NBlend, diet with normal protein content, based on casein and corn gluten meal; NCGM, diet with normal protein content, based on corn gluten meal; LCAS, diet with low protein content, based on casein; LBlend, diet with a low protein content, based on casein and corn gluten meal; LCGM, diet with low protein content, based on corn gluten meal.

**Figure 3:**
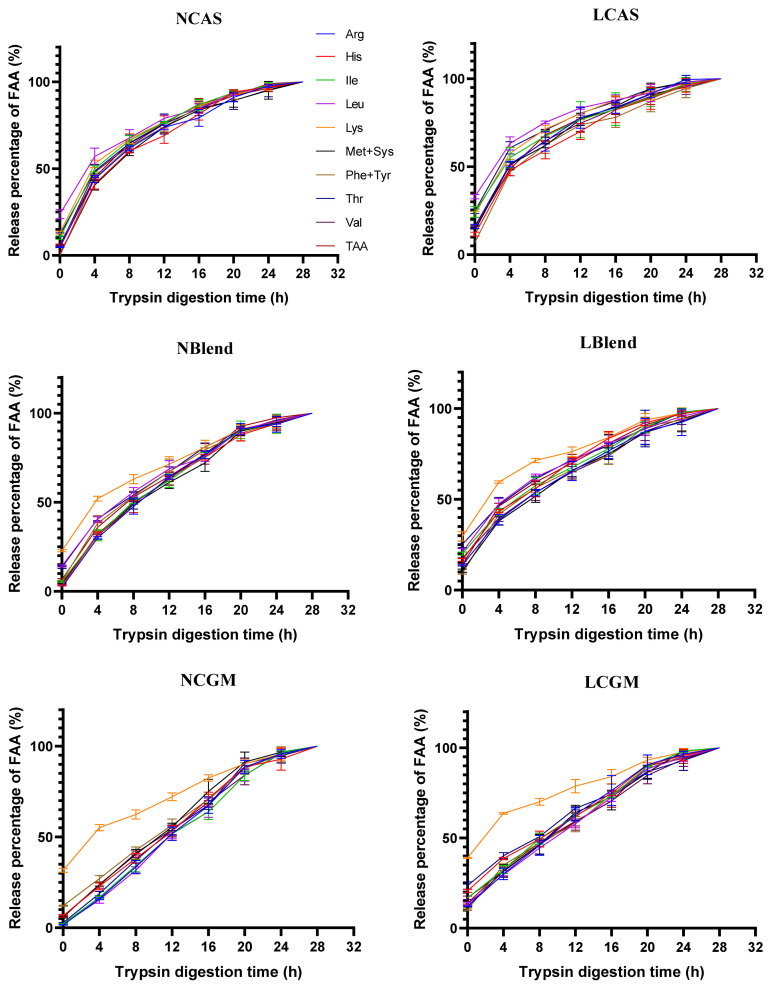
Percentage release of AA from diets over time. NCAS, diet with normal protein content, based on casein; NBlend, diet with normal protein content, based on casein and corn gluten meal; NCGM, diet with normal protein content, based on corn gluten meal; LCAS, diet with low protein content, based on casein; LBlend, diet with a low protein content, based on casein and corn gluten meal; LCGM, diet with low protein content, based on corn gluten meal; TAA, total free amino acids.

**Figure 4:**
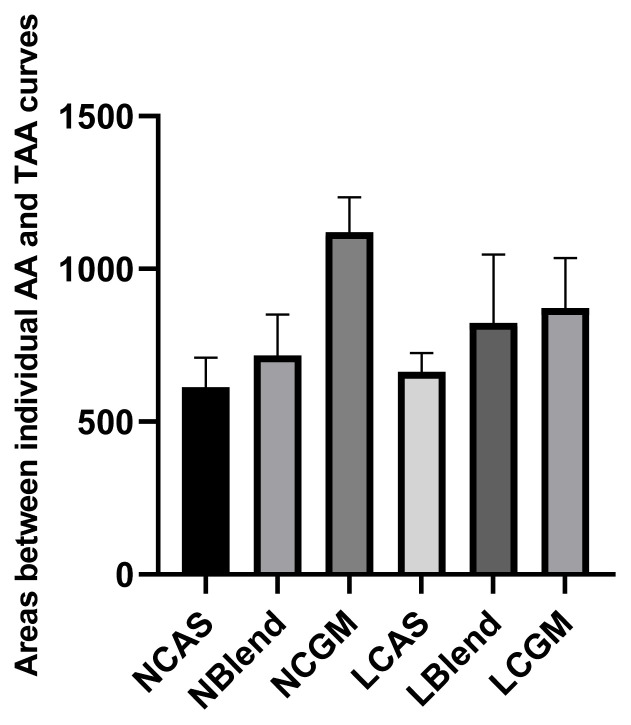
Absolute differences in the areas under the curves for the percentage release of individual free amino acids and the amino acids as a whole. NCAS, diet with normal protein content, based on casein; NBlend, diet with normal protein content, based on casein and corn gluten meal; NCGM, diet with normal protein content, based on corn gluten meal; LCAS, diet with low protein content, based on casein; LBlend, diet with a low protein content, based on casein and corn gluten meal; LCGM, diet with low protein content, based on corn gluten meal.

As shown in Figure 1, TFAA content in hydrolysate of normal protein diet was higher than that of low protein diet. As shown in Figure 2, in the CAS-containing diet and the mixed diet, the peak of FAA release content per 4 h was in the early stage of trypsin digestion, while in the CGM group, the peak of FAA release content per 4 h was in the late stage of trypsin digestion. As shown in Figure 3, there were different AA release percentage curves among different treatments. To quantify the disparities between AA release, we calculated the areas between the individual FAA and TFAA curves. As shown in Table 2 and Figure 4, the CAS-containing diet and the mixed diet had lower area between the curves compared with the CGM-containing diet (p<0.05). Protein content had no significant effect on area between the curves.

### Growth performance

As shown in Table 3, the CAS containing diet and the mixed diet had higher final body weight, ADG and G:F ratio compared with the CGM-containing diet (p<0.05). Diet with normal protein content had higher final body weight, ADG and G:F ratio than diet with low protein content (p<0.05).

### Nitrogen balance

As shown in Table 4, the CAS-containing diet and the mixed diet had higher nitrogen retention and nitrogen biological value compared with the CGM-containing diet (p<0.05). Diet with normal protein content had higher nitrogen loss and nitrogen retention and lower nitrogen digestibility, nitrogen retention, % intake, nitrogen biological value than diet with low protein content (p<0.05). There were interactions between protein source and protein content with respect to urine nitrogen content. The urine nitrogen content of the NCGM group was higher than that of the other groups (p< 0.01), but there was no difference between the NBlend and NCGM groups. The urine nitrogen content of the normal-protein groups was higher than that of the low-protein groups (p<0.01), but there were no differences among the three low-protein groups.

### Serum biochemical indices

As shown in Table 5, there were no differences in insulin concentration between the groups. Plasma urea nitrogen was higher in normal-protein diet than in low-protein diet and lower in CAS-containing diet and mixed diet than in CGM-containing diet (p<0.05).

### Expression of mediators of muscle synthesis and degradation

**Figure 5:**
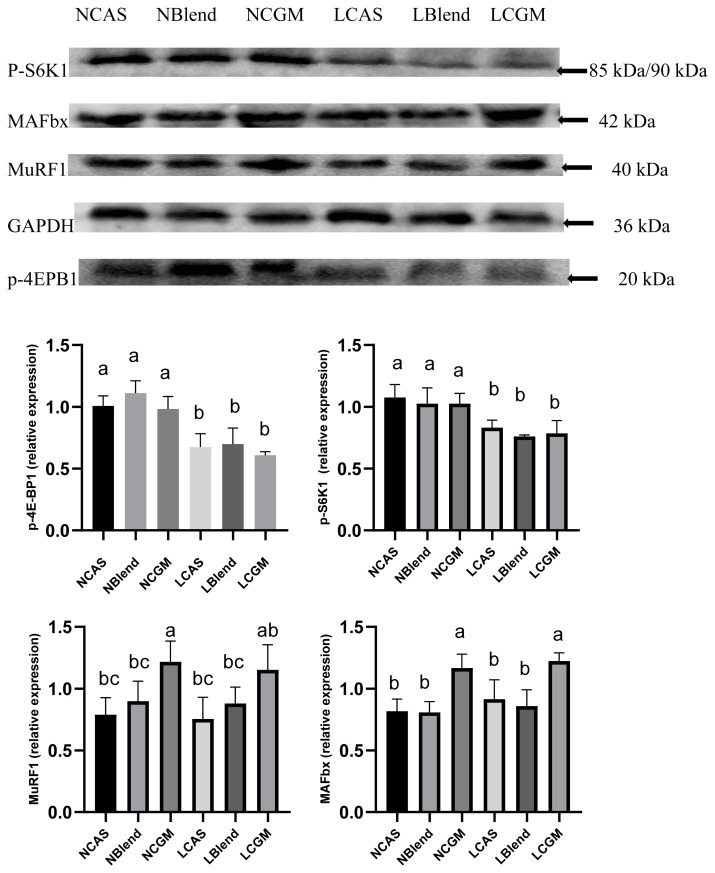
Protein expression of key mediators of muscle synthesis and degradation in each group. NCAS, diet with normal protein content, based on casein; NBlend, diet with normal protein content, based on casein and corn gluten meal; NCGM, diet with normal protein content, based on corn gluten meal; LCAS, diet with low protein content, based on casein; LBlend, diet with a low protein content, based on casein and corn gluten meal; LCGM, diet with low protein content, based on corn gluten meal; p-4E-BP1, phosphorylated elF 4E binding protein-1; p-S6K1, phosphorylated ribosomal protein S6 kinase; MuRF1, muscle ring finger 1; MAFbx, muscle atrophy F-box. ^a–c^ In each panel, different superscript letters represent significant differences (p<0.05).

As shown in Figure 5, the CAS-containing diet and the mixed diet had lower expression of MuRF1 and MAFbx compared with the CGM-containing diet (p<0.05). The p-4E-BP1 and p-S6K1 levels in the normal-protein diet groups were higher than in the low-protein diet groups (p<0.05).

## DISCUSSION

In the present study, we have shown that a 2.65% unit (SID) reduction in CP content significantly reduces the growth performance of piglets, in contrast to the results of numerous previous studies [3–5], on the basis of which the authors concluded that reductions of <4% unit CP did not affect growth performance. This difference may be explained by differences in the standards used. The “normal” protein content used in the present study was lower than NRC (1998) [15] requirement and the studies conducted, and in addition, the low-protein diet CP content was >5% unit lower than that of the “normal” diet. By contrast, Che et al [16] reported that the growth performance of piglets was decreased when the dietary protein content was reduced by 4% unit versus the NRC (2012) standard [7].

Even if sufficient levels of EAA are added, a low dietary protein concentration will still have adverse effects on piglets, which may be caused by deficiency of some conditionally essential amino acids (CEAA). Conditionally essential AA are defined as being able to be synthesized endogenously, but the efficiency of their synthesis is insufficient to keep pace with their utilization under particular circumstances, such as weaning, injury, and stress [17]. For piglets, the CEAA include Gln, Glu, Gly, and Pro, which play important roles in growth, development, and reproduction, and have been referred to as functional AA [18]. The phosphorylation of S6K1 and 4E-BP1, which are downstream intermediates in the mTOR signaling pathway, was higher in the muscle of pigs in the normal-protein diet groups than in pigs in the low-protein diet groups in the present study, which may be explained by deficiencies in NEAA. The mTOR signaling pathway is a key pathway in the regulation of protein synthesis, including in the regulation of expression of initiation and elongation factors, as well as of ribosome biogenesis itself, which can be activated by AA and growth factors, and inhibited by nutrient or energy deprivation [19]. Rezaei et al [20] showed that supplementation of a low-protein diet with Gln and Glu maintains the activation of translation initiation factors and optimal protein synthesis in young pigs. In addition, Hsu et al [21] showed that the addition of Gln to the diet improves villous morphology and the xylose absorption capacity of the small intestine, and thereby growth performance. Finally, Kirchgessner et al [22] showed that the addition of Pro increases the ADG and G:F and reduces the PUN of piglets in a dose-dependent manner.

Corn gluten meal is a byproduct of the maize industry that is rich in Leu [23] and can serve as an unconventional protein source for piglets [24,25]. Casein is a high-quality animal protein that is abundant in EAA, has a balanced AA composition, and has a true digestibility of almost 100% in pigs. Casein and CGM have different concentrations of AA and SID of AA, and therefore, to equalize the quantities of TFAA and individual FAA released from each in the GIT of the pigs, we used the SID to formulate the diets and supplemented them with the corresponding FAA.

The release of FAA in the intestine is faster than that of protein-derived AA [16,26]. The disparity in the CGM groups was often higher than in the CAS and Blend groups because the release of AA was slower from the CGM diets. Different protein sources have differing compositions and structures, which affects their solubility and digestibility [11,27]. Zein is present at a high concentration in CGM, and this contains numerous hydrophobic AA and S-S and O-H bonds that promote stable α-helical structure formation [12], both of which render zein poorly soluble in water. In addition, the poor digestibility of plant-based proteins in piglets may be due to incomplete development of digestive enzymes [24]. Abdallah et al [13] compared the *in vitro* digestion of SBM, fish meal, CGM, and fermented SBM over 28 h, and found that the FAA release from CGM diet was significantly lower than the release from the other diets at between 8 and 20 h, and did not reach the same level until 24 to 28 h, which indicates that AA are released more slowly from CGM. In the present study, we found that the peak AA release from CAS during trypsin digestion was within 0 to 4 h, whereas the peak AA release from CGM was at 16 to 20 h. The slow release of AA from CGM proteins, in contrast to the fast release of the added FAA, exaggerates the degree of AA imbalance associated with this diet. Conversely, CAS is rapidly hydrolyzed in piglet intestine [6], such that the imbalance was lower in the CAS groups.

In the present study, the CGM diets were associated with lower growth performance, nitrogen digestibility, and nitrogen retention, % intake than the CAS and Blend groups, which may have been related to the lower hydrolysis or dissolution rates of CGM. A similar conclusion was reached by Asche et al [24]. Even though the CGM, CAS, and Blend diets shared the similar SID AA, the differing digestion dynamics of the protein sources may have a significant effect on N availability. When AA are released asynchronously from a diet, particular AA may appear in large amounts in the small AA pool of the piglets, whereas others that are necessary for protein synthesis are present in insufficient quantities, resulting in the deamination of the excess. The higher concentrations of PUN and urinary N that were identified in the CGM groups in the present study are consistent with this explanation.

There were no differences in the phosphorylation of S6K1 or 4E-BP1 between the piglets fed the CAS, Blend, and CGM diets in the present study, but the expression of the E3 ligases MuRF1 and MAFBx, which form part of the UPP, was higher in the CGM groups than in the CAS or Blend groups. Skeletal muscle MAFbx and MuRF1 are generally considered to be the principal mediators of protein degradation, which is closely related to muscle atrophy [28]. We speculate that the rate of protein degradation in the CGM groups was higher to maintain muscle synthesis in skeletal muscle because of the deficiency of some AA. Luo et al [29] showed that growing rats fed zein had higher mRNA muscle expression of MuRF1 and MAFBx than those that were fed CAS or soy protein isolate. Moreover, the feeding of low-protein diets reduces protein intake, but more FAA are often added to such diets to meet the EAA requirements of the piglets, which may increase the imbalances in the AA supplied, resulting in poorer growth performance [26]. However, the imbalance in AA supply was similar in the low- and normal-protein versions of the CAS, Blend, and CGM diets in the present study, which may be because the TAA supply was also altered by the larger amounts of CAA that were added to the low-protein diets.

The fecal N content was higher and N utilization was lower in the CGM groups than in the CAS groups, which might be because the intake of N in the CGM groups was higher. To equalize the SID CP, CGM-based diets often have higher air-dried basic CP than CAS-based diets. Reducing N intake has been widely shown to reduce N loss and PUN, and increase N utilization in piglets [30,31]. However, these effects might be because of the suboptimal digestion of CGM. Casein is absorbed faster in the GIT and is associated with slower GIT emptying [25], which allows its component AA to be fully absorbed and utilized. However, AA release from CGM is highest in the distal small intestine; therefore, the AA cannot be efficiently absorbed [24]. Nevertheless, faster AA release is not necessarily better. Rivest et al [27] simulated porcine small intestinal digestion and showed that the absorptive capacity of the small intestine limits its absorption of AA. Therefore, excess provision of AA may lead to the upper absorption limit of the intestine being exceeded, resulting in AA loss.

Studies of the effects of exercise on muscle synthesis in humans and the relationship with the rate of digestion of ingested proteins have demonstrated that the consumption of rapidly digested proteins, such as whey, causes a rapid, temporary rise in plasma AA concentrations, but this also results in the oxidation and loss of excess AA, whereas the consumption of slowly digested proteins, such as CAS and soybean protein, is associated with modest but more sustained increases in plasma AA concentrations [10,32]. Exercise promotes the utilization of AA by skeletal muscle, and early-weaning piglets are a good model of the utilization of AA because of the fast-growing nature of piglet muscle [33]. Reidy et al [10] suggested that a mixture of proteins could combine the advantages of rapidly digested proteins with those of more slowly digested proteins, which would sustain the phosphorylation of S6K1 and the peak fractional synthetic rate for a longer period of time after the appearance of hyperammonemia. However, there were no significant differences in growth performance, N utilization, or protein expression of mTOR and components of the UPP between the CAS and Blend groups in the present study, which may be attributed to their similar AA content. Although CAS was found to release AA more slowly than whey in the previous study [10,32], CAS had higher digestibility than CGM in the present study. In addition, the previous studies described above were conducted in humans who ingested single meals of each protein, and there were few long-term feeding trials, which would be more similar to the present study.

The induction of translation by insulin and AA in the postprandial skeletal muscle of piglets is a key factor in the rapid neonatal growth of muscle [34]. In the present study, there were no significant differences in plasma insulin between the groups. Therefore, the differences in growth performance and nitrogen deposition may be caused by the differing availability of AA [33]. Atinmo et al [35] showed that limiting the protein content of a diet reduces the insulin concentrations of piglets. In addition, Deng et al [2] fed piglets a low-protein diet supplemented with EAA, and showed that the insulin concentration did not significantly differ from that of the control group, which implies that insulin secretion may be affected by the EAA supply.

## CONCLUSION

In summary, there were no differences in nitrogen deposition or growth performance in piglets between the CAS and Blend groups. By contrast, piglets fed the CGM-based diets showed lower nitrogen retention, and % intake, probably because of a relative imbalance in AA release. We conclude that moderate and steady release of AA, whether from a protein-limited diet or not, is beneficial for the maximization of nitrogen deposition. However, further work is needed to confirm this finding and to identify the most critical growth periods.

## Figures and Tables

**Table 1:** Ingredients and calculated nutrient composition of experimental diet (as-fed basis)

Items	Diet					

	NCAS	NBlend	NCGM	LCAS	LBlend	LCGM
Ingredient (%)						
Corn	64.02	58.03	52.05	68.47	63.18	57.90
Wheat bran	2.00	2.00	2.00	2.00	2.00	2.00
Soybean meal	17.40	18.38	19.35	12.28	14.28	16.27
Casein	7.07	3.54	-	6.00	3.00	-
Corn gluten meal	-	7.34	14.68	-	5.27	10.54
Sucrose	3.00	3.00	3.00	3.00	3.00	3.00
Soybean oil	1.10	2.16	3.21	1.17	2.13	3.10
L-Arg, 99.7%	0.06	0.03	-	0.20	0.18	0.16
L-Ile, 100.0%	0.09	0.05	-	0.22	0.19	0.16
L-Leu, 99.2%	0.79	0.40	-	1.00	0.72	0.44
L-Cys, 99.3%	-	0.03	0.06	0.09	0.11	0.13
L-Tyr, 99.9%	-	0.21	0.41	0.24	0.30	0.36
L-His, 99.5%	-	0.02	0.04	0.07	0.10	0.12
L-Lys.HCL, 78.8%	0.28	0.45	0.61	0.48	0.60	0.73
DL-Met, 99.5%	0.01	0.03	0.05	0.07	0.09	0.11
L-Phe, 100.0%	-	0.23	0.45	0.26	0.33	0.39
L-Thr, 99.5%	0.12	0.12	0.11	0.22	0.22	0.22
L-Trp, 99.5%	0.01	0.02	0.04	0.04	0.05	0.06
L-Val, 99.3%	0.05	0.04	0.03	0.16	0.16	0.17
Limestone	1.00	1.01	1.02	0.88	0.99	1.09
Dicalcium phosphate	1.15	1.10	1.05	1.18	1.18	1.19
Salt	0.85	0.84	0.84	0.85	0.85	0.84
Premix^1)^	1.00	1.00	1.00	1.00	1.00	1.00
Nutrient levels (%)						
Net energy (MJ/kg)	10.26	10.26	10.25	10.25	10.26	10.26
Crude protein	19.79	21.23	22.95	17.08	17.84	18.36
Crude protein (SID)	17.54	17.54	17.53	14.89	14.89	14.88
Arg (SID)	1.06	1.06	1.06	1.07	1.07	1.07
His (SID)	0.49	0.49	0.49	0.48	0.48	0.48
Ile (SID)	0.85	0.85	0.85	0.85	0.85	0.85
Leu (SID)	2.43	2.43	2.42	2.43	2.43	2.43
Lys (SID)	1.35	1.35	1.35	1.35	1.35	1.35
Met+Cys (SID)	1.24	1.24	1.24	1.24	1.24	1.24
Phe+Tyr (SID)	3.26	3.26	3.25	3.25	3.25	3.25
Thr (SID)	0.79	0.79	0.79	0.79	0.79	0.79
Trp (SID)	0.22	0.22	0.22	0.22	0.22	0.22
Val (SID)	0.94	0.93	0.91	0.86	0.86	0.86
Crude fiber	1.73	1.74	1.75	1.63	1.67	1.70
Ca	0.80	0.80	0.80	0.80	0.80	0.80
Available P	0.40	0.40	0.40	0.40	0.40	0.40

NCAS, diet with normal protein content, based on casein; NBlend, diet with normal protein content, based on casein and corn gluten meal; NCGM, diet with normal protein content, based on corn gluten meal; LCAS, diet with low protein content, based on casein; LBlend, diet with a low protein content, based on casein and corn gluten meal; LCGM, diet with low protein content, based on corn gluten meal; SID, standardized ileal digestible.

1) Supplied the following per kilogram of diet: vitamin A, 5,000 IU; vitamin D_3_, 500 IU; vitamin E, 20 mg; vitamin K, 2 mg; vitamin B_6_, 4 mg; vitamin B_12_, 4 mg; vitamin B_1_, 1.5 mg; biotin, 0.10 mg; folic acid, 0.50 mg; nicotinic acid, 25 mg; pantothenic acid, 2 mg; riboflavin, 4 mg; oxytetracycline, 50 mg; antioxidant, 100 mg; Fe, 200 mg as FeSO_4_; Cu, 20 mg as CuSO_4_; Mn, 0.10 mg as MnSO_4_; Co, 0.10 mg as CoCO_3_; Zn, 250 mg as ZnO; I, 0.5 mg as KIO_3_; Se, 1 mg as Na_2_SeO_3_.

**Table 2:** Absolute differences in the areas under the curves for the percentage release of individual free amino acids and the amino acids as a whole (n = 3)

Parameter	Protein source			Protein content		Diet						SEM	p-value		
			
	CAS	Blend	CGM	Normal	Low	NCAS	NBlend	NCGM	LCAS	LBlend	LCGM		Source	Content	S×C
Area	638^b^	770^b^	996^a^	817	786	613	717	1121	664	823	872	33.41	<0.01	0.657	0.107

NCAS, diet with normal protein content, based on casein; NBlend, diet with normal protein content, based on casein and corn gluten meal; NCGM, diet with normal protein content, based on corn gluten meal; LCAS, diet with low protein content, based on casein; LBlend, diet with a low protein content, based on casein and corn gluten meal; LCGM, diet with low protein content, based on corn gluten meal; SEM, standard errors of the mean.

a,b Within the same row, different superscript letters represent significant differences with respect to protein content, source, or both (p<0.05).

**Table 3:** Growth performance of piglets fed diets with differing protein content and source (n=4)

Parameter	Protein source			Protein content		Diet						SEM	p-value		
			
	CAS	Blend	CGM	Normal	Low	NCAS	NBlend	NCGM	LCAS	LBlend	LCGM		Source	Content	S×C
Initial BW (kg)	7.49	7.46	7.42	7.45	7.47	7.48	7.49	7.38	7.50	7.43	7.47	0.087	0.947	0.932	0.942
Final BW (kg)	16.78^a^	16.68^a^	15.96^b^	17.07	15.87	17.43	17.25	16.53	16.12	16.12	15.38	0.108	0.012	<0.01	0.941
ADFI (g/d)	503	500	494	501	497	503	501	498	502	499	490	2.179	0.297	0.406	0.743
ADG (g/d)	344^a^	342^a^	316^b^	356	311	368	362	339	319	322	293	3.139	<0.01	<0.01	0.839
G:F ratio	0.684^a^	0.683^a^	0.639^b^	0.711	0.626	0.732	0.721	0.680	0.636	0.645	0.598	0.005	<0.01	<0.01	0.692

NCAS, diet with normal protein content, based on casein; NBlend, diet with normal protein content, based on casein and corn gluten meal; NCGM, diet with normal protein content, based on corn gluten meal; LCAS, diet with low protein content, based on casein; LBlend, diet with a low protein content, based on casein and corn gluten meal; LCGM, diet with low protein content, based on corn gluten meal; SEM, standard errors of the mean; BW, body weight; ADFI, average daily feed intake; ADG: average daily gain; G:F ratio: gain-to-feed ratio.

a,b Within the same row, different superscript letters represent significant differences with respect to protein content, source, or both (p<0.05).

**Table 4:** Nitrogen balance of piglets fed diets with differing protein content and source (n = 4)

Parameter	Protein source			Protein content		Diet						SEM	p-value		
			
	CAS	Blend	CGM	Normal	Low	NCAS	NBlend	NCGM	LCAS	LBlend	LCGM		Source	Content	S×C
Nitrogen intake (g/d)	25.76^c^	26.53^b^	27.38^a^	29.23	23.88	28.25	29.11	30.34	23.27	23.95	24.42	0.116	<0.01	<0.01	0.236
Fecal nitrogen (g/d)	3.87^c^	4.57^b^	5.79^a^	5.79	3.70	4.74	5.65	6.96	3.00	3.48	4.62	0.069	<0.01	<0.01	0.218
Urine nitrogen (g/d)	4.58^b^	4.65^b^	4.92^a^	5.66	3.77	5.47^b^	5.49^b^	6.02^a^	3.70^c^	3.80^c^	3.82^c^	0.04	<0.01	<0.01	0.037
Nitrogen loss (g/d)	8.46^c^	9.21^b^	10.71^a^	11.45	7.48	10.21	11.14	12.98	6.70	7.29	8.44	0.082	<0.01	<0.01	0.054
Nitrogen retention (g/d)	17.30^a^	17.32^a^	16.67^b^	17.79	16.40	18.03	17.97	17.36	16.57	16.67	15.98	0.091	0.014	<0.01	0.934
Nitrogen digestibility (%)	85.15^a^	83.04^b^	79.06^c^	80.29	84.55	83.21	80.60	77.06	87.09	85.48	81.07	0.198	<0.01	<0.01	0.546
Nitrogen retention, % intake (%)	67.51^a^	65.66^b^	61.33^c^	60.93	68.74	63.85	61.72	57.22	71.18	69.6	65.43	0.239	<0.01	<0.01	0.749
Nitrogen biological value (%)	79.22^a^	79.00^a^	77.49^b^	75.85	81.29	76.73	76.59	74.25	81.72	81.42	80.72	0.21	<0.01	<0.01	0.238

NCAS, diet with normal protein content, based on casein; NBlend, diet with normal protein content, based on casein and corn gluten meal; NCGM, diet with normal protein content, based on corn gluten meal; LCAS, diet with low protein content, based on casein; LBlend, diet with a low protein content, based on casein and corn gluten meal; LCGM, diet with low protein content, based on corn gluten meal; SEM, standard errors of the mean.

a–c Within the same row, different superscript letters represent significant differences with respect to protein content, source, or both (p<0.05).

**Table 5:** Plasma insulin and urea nitrogen concentrations of piglets fed diets with differing protein content and source (n = 4)

Parameter	Protein source			Protein content		Diet						SEM	p-value		
			
	CAS	Blend	CGM	Normal	Low	NCAS	NBlend	NCGM	LCAS	LBlend	LCGM		Source	Content	S×C
Insulin (mmol/L)	42.30	43.64	40.91	43.56	41.00	44.33	45.52	40.82	40.26	41.75	41.00	1.156	0.636	0.283	0.709
Plasma urea nitrogen (mIU/L)	4.08^b^	4.16^b^	4.81^a^	4.67	4.02	4.43	4.44	5.15	3.72	3.87	4.46	0.120	0.045	0.014	0.965

NCAS, diet with normal protein content, based on casein; NBlend, diet with normal protein content, based on casein and corn gluten meal; NCGM, diet with normal protein content, based on corn gluten meal; LCAS, diet with low protein content, based on casein; LBlend, diet with a low protein content, based on casein and corn gluten meal; LCGM, diet with low protein content, based on corn gluten meal; SEM, standard errors of the mean.

a,b Within the same row, different superscript letters represent significant differences with respect to protein content, source, or both (p<0.05).
